# Effects of different sources of carbohydrates on intake, digestibility, chewing, and performance of Holstein dairy cows

**DOI:** 10.1186/2049-1891-5-6

**Published:** 2014-01-13

**Authors:** Simin Poorkasegaran, Asadollah Teimouri Yansari

**Affiliations:** 1 Department of Animal Science, Animal Science and Aquaculture Faculty, Sari Agricultural and Natural Resource University, Sari, Mazandaran, Iran; 2 Department of Animal Science, Educational Center of Tajan, GhaemShahar, Mazandaran, Iran

**Keywords:** Chewing activity, Dairy cow, Detergent-soluble carbohydrate, Fiber, Neutral detergent fiber, Physically effective fiber, Ruminal characteristics

## Abstract

To investigate the effects of different sources of carbohydrates on intake, digestibility, chewing, and performance, nine lactating Holstein dairy cows (day in milk= 100±21 d; body weight=645.7 ± 26.5 kg) were allotted to a 3 × 3 Latin square design at three 23-d periods. The three treatments included 34.91% (B), 18.87% (BC), and 18.86% (BB) barley that in treatment B was partially replaced with only corn or corn plus beet pulp in treatments BC and BB, respectively. The concentration of starch and neutral detergent soluble carbohydrate varied (22.2, 20.2, and 14.5; 13.6, 15.9, and 20.1% of DM in treatments B, BC, and BB, respectively). Cows in treatment BB showed a higher DMI and improved digestibility of DM, NDF, and EE compared with treatments B or BC. Ruminal pH was higher in cows fed on BB (6.83) compared with those that received B or BC treatments (6.62 and 6.73, respectively). A lower proportion of propionate accompanied the higher pH in the BB group; however, a greater proportion of acetate and acetate: propionate ratio was observed compared with cows fed either on the B or BC diet. Moreover, cows fed on the BB diet showed the lowest ruminal passage rate and longest ruminal and total retention time. Eating time did not differ among treatments, rumination time was greater among cows fed on the BB diet compared with the others, whereas total chewing activity was greater than those fed on BC, but similar to those fed on B. The treatments showed no effect on milk yield. Partially replacing barley with corn or beet pulp resulted in an increase in milk fat and a lower protein concentration. Changing dietary NFC with that of a different degradability thus altered intake, chewing activity, ruminal environment, retention time or passage rate, and lactation performance. The results of this study showed that beet pulp with a higher NDF and a detergent-soluble carbohydrate or pectin established a more consistent ruminal mat than barley and corn, thus resulting in higher mean retention time and chewing activity, whereas no changes in 3.5% FCM and milk fat were observed.

## Background

Three components affect the balance of carbohydrates in the diets of lactating dairy cattle. The first two are the nature of the NDF and the nature of non-fiber carbohydrates (NFC), and the third component is one of the following: NDF content, NFC content, or NDF:NFC ratio [1-3]. Carbohydrates consisting of NDF and NFC fractions make up 65%–75% of rations. However, issues have been associated with the use of an NDF because this component ranges from 27% to 37% in dietary DM, whereas NFC content ranges from 45% to 35% [3,4]. The NFC has been represented as a single value for feeds or diets, but the type of carbohydrates in this fraction largely varies. For example, the NFC in corn grain is mostly starch (65%–70% of DM), beet and citrus pulp provides sugar (12%–40% of DM) and neutral detergent-soluble carbohydrate (NDSC) (25%–44% of DM), and sugar (mono- and oligosaccharides) predominates in molasses [5]. In addition, some of the variation in the results of the effect of the type and amount of NFC on high-producing lactating cows may be related to: 1) the effects of rapidly degradable starch on ruminal digestion of fiber; 2) the level to which NFC replaces fiber in the ration; 3) the site of starch digestion; 4) the level of DMI and the physiological state of the animal; and 5) the conservation and processing methods of NFC sources used to alter the rate and the extent of NFC digestion [4-6]. However, the optimal concentration of NFC in dairy cow diets has not been well defined. The lack of clear recommendations may be at least due to the wide compositional and nutritional variation in the sources of NFC, the kinetics of ruminal digestion, and the yields of metabolizable nutrients [4]. Therefore, improving the understanding of the digestion and metabolism of various NFC types that are fed to dairy cattle can improve animal performance and profitability while maintaining health.

The physically effective NDF (peNDF) incorporates information on particle length, NDF content of the diet, and impact of replacing forage fiber with NFC based on the use of small particles of byproducts and measuring the animal’s chewing activity [7,8]. However, this concept does not account for different sources of NFC or differences in fermentability among various feeds. The nature of NFC differs among feeds and diets. In evaluating the effectiveness of the NDF approach, the effect of removing or adding NFC has been entirely attributed to NDF [1,2,9]. Based on this simple assumption, the effectiveness of NDF from all sources is thus based on the replacement of NFC. Therefore, understanding the nutritional value of each NFC type may aid in the use of available feeds, including byproducts, to efficiently formulate dairy cow diets that would enhance production and health. However, extensive information on the optimal level of starch and digestible fiber in rations for dairy cows is warranted to facilitate in the design of strategies to prevent ruminal acidosis and the efficient conversion of energy into milk. The objective of this trial was to compare the effects of three different carbohydrate sources, namely barely, corn, and beet pulp (BP), on stimulating intake, digestibility, chewing activity, ruminal characteristics, and early lactation in Holstein dairy cows.

## Materials and methods

### Animals and diets

The experiment was conducted at the Dairy Farm of the Agricultural Education and Research Centre of Jehad-e-Keshavarzi, Sari, Iran. Nine multiparous mid-lactation Holstein dairy cows (BW = 645.7 ± 26.5 kg; DIM = 100 ± 21 d) were subjected to a 3 × 3 Latin square design. The experiment consisted of three 23-d periods (adaptation, 14 d; sample collection, 7 d; and measurement of chewing activity, 2 d). The three treatments containing 34.91% (B), 18.87% (BC), and 18.86% (BB) barley as DM were used. The barley in treatment B was partially replaced with only corn or corn plus beet pulp in treatments BC and BB, respectively (Table 1). Barley grain, corn grain, and BP were prepared by milling normal BP using a 2-mm screen pore size miller. Diets were similar in chemical (16% CP and 6.65 MJ/kg of NEL) and physical (geometric mean and peNDF) characteristics (Table 2). Cows were housed in tie-stalls and fed *ad libitum* twice daily at 0900 and 2100 h with TMR, allowing for at least 10% residual (as-fed basis). Water and mineralized salt stone were available for cows during the experiment.

**Table 1:** Ingredients and chemical composition of three total mixed rations containing three different sources of non-fiber carbohydrates

**Item**	**Treatments**^**1**^			**SEM**	***P *** **value**
	**B**	**BC**	**BB**		
Ingredients, % of DM^2^					
Alfalfa hay	23.41	23.69	23.57		
Wheat straw	15.53	15.84	15.8		
Barley grain	34.91	18.87	18.86		
Corn grain	…	15.89	…		
Beet pulp	…	…	15.89		
Cotton seed meal	11.19	8.88	8.90		
Soybean meal	3.01	2.99	3.01		
Wheat bran	8.01	9.68	9.69		
Sunflower meal	2.69	2.99	3.01		
Calcium carbonate	0.43	0.4	0.5		
Calcium phosphate	0.25	0.20	0.20		
Salt	0.10	0.10	0.10		
Vitamin supplement^3^	0.25	0.25	0.25		
Urea	0.22	0.22	0.22		
Chemical compositions, % of DM					
CP	16.2	15.9	15.9	0.232	0.321
NDF	36.2	36.9	37.7	0.864	0.079
ADF	23.1	22.6	24.6	0.541	0.092
NFC	38.9	38.9	37.9	0.682	0.061
Starch	22.2^a^	20.2^a^	14.5^b^	0.792	0.001
Sugar	3.1	2.8	3.3	0.432	0.213
NDSC^4^	13.6^c^	15.9^b^	20.1^a^	0.232	0.001
EE	2.3	2.4	2.4	0.219	0.212
Ash	6.4	5.9	6.1	0.672	0.431
NEl^5^, Mcal/kg of DM	1.58	1.59	1.58	0.232	0.452

^a, b, c^Means within a row with different subscripts differ (*P* < 0.05).

^1^Treatments were diets containing barley + basal diet (B), barley and corn + basal diet (BC), and barley and beet pulp + basal diet (BB).

^2^Alfalfa, wheat straw, barley grain, corn, beet pulp, cotton seed meal, soy bean meal, wheat bran, and sun flower meal (DM basis) were chemically composed of 89.1%, 50.9%, 18.7%, and 15.3%; 91.2, 75.2, 10.4, and 3.4; 93.2, 22.1, 55.4, and 10.5; 96.7, 11.0, 78.3, and 8.5; 93.4, 44.4, 41.0, and 9.0; 90.7, 45.2, 16.3, and 27.3; 90.3, 17.8, 24.7, and 42.5; 90.3, 52.3, 24.4, and 16.5; and 92.0, 55.7, 17.3, and 16.1 for OM, NDF, NFC, and CP, respectively.

^3^Contained 40% NaCl, 25% Dyna mate, 10% K, 7% Mg, 12% S, 1,000 mg Fe/kg, 2% ZnSO_4_.H_2_O, 2% MnSO_4_.4H_2_O, 0.01% CoSO_4_.6H_2_O, 0.009% Na_2_SeO_3_, 0.012% ethylenediamine dihydroiodide, 0.8% CuSO_4_.5H_2_O, 680,000 IU/kg of Vitamin A, 160,000 IU/kg of Vitamin D, and 2,000 IU/kg of Vitamin E.

^4^NDSC = Neutral detergent soluble carbohydrate.

^5^NEl was calculated based on NRC [4].

**Table 2:** Physical characteristics of three total mixed rations containing three different sources of non-fiber carbohydrate

**Item**	**Treatments**^**1**^			**SEM**	***P *** **value**
	**B**	**BC**	**BB**		
Particle size distribution (material retain on each sieve, % of DM)					
19 mm	9.7	10.1	9.8	0.732	0.213
8 mm	22.2	22.1	20.4	0.912	0.133
1.18 mm	34.4	34.2	36.9	1.648	0.422
Pan	33.7	33.6	32.9	0.641	0.455
Geometric mean, mm	3.85	3.92	3.80	0.692	0.087
Standard deviation of geometric mean	1.20	1.24	1.34	0.106	0.093
Physical effective factor					
pef_m_^2^	66.2	66.3	66.9	0.509	0.101
pef _> 8_^3^	34.4	34.6	31.3	0.322	0.288
pef _> 1.18_^4^	66.3	66.4	67.1	0.694	0.083
Physical effective NDF, % of DM					
peNDF_m_	24	24	26	1.13	0.063
peNDF _> 8_	12	13	12	1.03	0.054
peNDF _> 1.18_	24	24	26	1.14	0.063

^1^Treatments were diets containing barley + basal diet (B), barley and corn + basal diet (BC), and barley and beet pulp + basal diet (BB).

^2^pef_m_ = physically effective factor, based on DM retained on sieve 1.18 mm [13].

^3^pef_>8_ = physically effective factor, determined as the proportion of DM retained on sieves of the original version of Penn State particle separator [10].

^4^pef_>1.18_ = physically effective factor, determined as the proportion of DM retained on sieves of the new version of Penn State particle separator [11].

^5^The peNDF was calculated by multiplying NDF content of each portion on each sieve on each pef [13].

### Particle length and effectiveness fiber

Feed particle size and distribution were determined by dry sieving in four replicates using two sets of a Penn State particle separator [10,11]. The NDF content of all materials retained on each sieve of the Penn State particle separator were measured [12]. Using three systems, the physical effective factor (**pef**) of the TMRs was determined. According to Mertens [13], Lammers et al. [10] and Kononoff [11], the pef was determined based on the proportion of DM retained on the 1.18-m sieve (**pef**_**m**_), on two 19- and 8-mm sieves (**pef**_**>8**_**)**, and three 19-, 8-, and 1.18-mm sieves (**pef**_**>1.18**_), respectively. The peNDF_m_**,** peNDF_>8_, and peNDF_>1.18_ were calculated by multiplying the NDF content of each portion on each sieve with the pef_m_**,** pef_>8_, and pef_>1.18_, respectively (Table 2).

### Body weight, intake, and apparent total-tract nutrient digestibility

The BW of cows was determined each wk. The DMI of the cows was measured daily (Table 3). Daily samples of TMRs, residuals and. total feces were collected daily for 5 d of each period at 0700 h on d 14 to 0700 h on d 20, weighed, and d 5 samples were composited and subsampled. The samples were dried at 55°C for 48 h and ground using a Wiley mill (1-mm screen), analyzed for DM, Kjeldahl N, ether extract, OM, ash at 605°C for 3 h [14], NDF, and ADF [12; with α-amylase being added for concentrates during NDF extraction; sodium sulfite was not added]. Neutral detergent fiber was expressed without residual ash. Non-fibrous carbohydrate was calculated using the following equation: 100 - % CP +% NDF +% ash +% EE [4]. The TMR composites were analyzed for soluble sugars using sucrose as the standard and for starch [5]. The proportion of NDSC was computed as NFC (%) using the following equation: [(Sugar (%) + Starch (%)] [5]. Using the chemical components of TMRs and feces, the intake and digestibility of nutrients were calculated. Intake (kg/d and% of DMI) of peNDF_m_, peNDF_>8_^,^ and peNDF_>1.18_ were calculated for each treatment (Table 3).

**Table 3:** Body weight, intake, and digestibility of nutrients of three total mixed rations containing three different sources of non-fiber carbohydrates

**Item**	**Treatments**^**1**^			**SEM**	***P *** **value**
	**B**	**BC**	**BB**		
BW, kg	600.63	591.84	583.25	11.212	0.140
Intake, kg/d					
DM	21.14^b^	21.02^b^	22.32^a^	0.530	0.004
NDF	7.65^b^	7.76^b^	8.57^a^	0.021	0.001
peNDF_m_^2^	5.10^b^	5.14^b^	5.74^a^	0.034	0.025
peNDF _> 8_^3^	2.63	2.68	2.68	0.231	0.075
peNDF _> 1.18_^4^	5.07^b^	5.15^b^	5.75^a^	0.023	0.024
CP	3.42	3.34	3.55	0.456	0.133
NFC	8.22	8.18	8.46	0.345	0.309
Starch	4.69^a^	4.25^a^	3.24^b^	0.431	0.002
Sugar	0.66	0.59	0.73	0.084	0.064
NDSC^5^	2.88^c^	3.34^b^	4.49^a^	0.104	0.001
EE	0.49	0.50	0.54	0.121	0.087
OM	19.79	19.78	20.96	0.961	0.342
Digestibility, %					
DM	64.58^b^	65.01^b^	66.91^a^	0.824	0.004
NDF	61.58^b^	62.38^b^	68.82^a^	1.028	0.004
ADF	51.67	52.21	52.33	0.769	0.100
CP	77.33	77.42	76.89	0.571	0.532
NFC	83.36^a^	80.37^b^	80.90^b^	1.424	0.032
EE	62.44^b^	66.52^ab^	67.33^a^	0.277	0.024
OM	64.68	64.61	63.53	1.143	0.200

^a, b, c^Means within a row with different subscripts differ (*P* < 0.05).

^1^Treatments were diets containing barley + basal diet (B), barley and corn + basal diet (BC), and barley and beet pulp + basal diet (BB).

^2^peNDF_m_ = physically effective NDF based on DM retained on sieve 1.18 mm [13].

^3^peNDF_>8_ = physically effective NDF, determined as the proportion of DM retained on sieves of the original version of Penn State particle separator [10].

^4^peNDF_>1.18_ = physically effective NDF determined as the proportion of DM retained on sieves of the new version of Penn State particle separator [11].

^5^NDSC = Neutral detergent soluble carbohydrate.

### Rumen fermentation and kinetics

On d 21 of each period, ruminal fluid samples (50 mL) from the ventral sac were collected 3 h after the morning feeding using the rumenocentesis technique. Ruminal pH was measured immediately after ruminal fluid collection using a pH meter (model 632, Metrohm, Herisau, Switzerland), and samples were frozen at −20°C. The concentration of ammonia nitrogen (NH_3_-N) was measured using a Kjeltec Auto Analyzer (Model 1030, Tecator Co. Sweden). Ruminal fluids were acidified by mixing with 2.5 mL of 6 mol/L HCl and then frozen for further analysis of VFA and NH_3_-N. Ruminal fluid was centrifuged at 25,000 × *g* for 20 min before measurement of VFA by gas chromatography (4% carbomax 80/120 BDA column, Supelco Inc., Bellefonte, PA; Autosystem XL gas chromatograph, Perkin Elmer Inc., Norwalk, CT) [15].

Digestive kinetics were measured using Cr-mordanted NDF alfalfa that was prepared by repeatedly soaking a mixture of alfalfa hay in dilute neutral detergent and rinsing until the NDF content of the material exceeded 80%. The fiber was then dried at 55°C. Chromium (Cr)- mordanted alfalfa NDF was prepared as previously described by Uden et al. [16], as the single-dose marker for solid passage rate measurement. Cr-mordanted fiber was prepared by mordanting alfalfa NDF ground through a 5-mm screen using a Wiley mill. Each cow was orally fed with a mix of 2 kg of concentrate and 250 g of marker before morning feeding on the 15^th^ d of each period. Fecal grab samples were collected at 0, 6, 10, 12, 14, 18, 22, 26, 30, 36, 42, 48, 54, 60, 72, 84, 96, 120, and 144 h after dosing from the rectum to determine the passage rate, ruminal, and total mean retention time, and time delay (transit time) of the marker. Samples were dry-ashed, and fecal Cr concentrations were determined by direct current plasma emission spectroscopy [14]. Fecal Cr excretion curves were fitted to the double compartment model, as represented by the following two exponential constants and a time delay [17]:

$$\begin{array}{l} Y=Ae_{1}^{k\left(t‒\mathrm{TT}\right)}-Ae_{2}^{‒k}\;\left(t‒\mathrm{TT}\right),k_{1}=k_{2}\;\mathrm{for}\;t^{3}T;Y=0\;\mathrm{for}\;t\\ \;=\mathrm{TT}; \end{array}$$

in which Y = marker concentration (ppm); A = scale parameter; k_1_ = ruminal passage rate (%/h); k_2_ = lower digestive tract passage rate (%/h); t = sampling time post dosing (h); and TT = transit time or time delay of marker. The total mean retention time was calculated as the sum of ruminal mean retention time (1/k_1_), and for the lower digestive tract, mean retention time (1/k_2_) plus the transit time. Parameters were estimated by NLIN regression using the PROC NLIN (iterative Marquardt method) of SAS® [18]. The estimated parameters were analyzed according to a previously described experimental design.

### Eating, ruminating, and chewing times

Eating and ruminating activities were monitored visually for all cows in each treatment over a 24-h period for 2 d and at d 22 to 23 of each period. Eating and ruminating activities were noted at 5-min intervals and each activity was assumed to persist for the entire interval. A period of rumination was defined as at least 5 min of ruminating activity, followed by at least 5 min without ruminating activity. Total time spent on chewing was calculated as the total time spent eating and ruminating [8].

### Milk production

Milk yield was recorded daily during the course of the experiment. On d 18 to 23 of each period, milk samples were collected each morning and evening. Approximately 100 mL of each milk sample were composited and analyzed for total protein, fat, and lactose [14].

### Statistical analysis

The data on particle size were analyzed as a completely randomized design for the effects of the diets and the two methods of particle size measurement using the REML variance component and PROC MIXED of SAS® [18]. Mean separation was determined using the PDIFF procedure and significance was declared at *P* < 0.05. The geometric mean and its standard deviation were calculated using ASAE S424.1 [7].

Using PROC MIXED of SAS® [18], the experimental data were analyzed in a 3 × 3 replicated Latin square design using following model:

$$Y_{\mathrm{ijkl}}=\mu +\mu_{i}+S_{j}+\mathrm{Co}w_{k\left(j\right)}+\mathrm{period}+e_{\mathrm{ijkl}}$$

in which Y_ijkl_ is the dependent variable_;_ μ is overall mean; T_i_ is fixed effect of the treatments (i = 1, 2, and 3 for diets containing B, BC, and BB); S_j,_ random effect of square (j = 1, 2, and 3); Cow_k(j)_, effect of cow within square; period_l_ within square, and e_ijkl_ is residual error. Means were separated using Duncan's multiple range tests, with an alpha level of 0.05.

## Results

### Nutrient composition, particle size distribution, and physical effectiveness of different treatments

Except for starch (*P =* 0.001) and NDSC (*P =* 0.001) content, the three TMRs showed similar chemical compositions and physical characteristics (Tables 1 and 2). Concentrations of starch were similar for treatments B and BC and both were greater than that observed with treatment BB (22.2, 20.2, and 15.9%, respectively). In addition, the concentration of NDSC increased with partial replacement of barley by corn and to a greater extent by partial replacement with BP (13.6, 15.9, and 20.1% for treatments B, BC, and BB, respectively). Distribution of particles, expressed as the percentage of total mass, retained on the 19, 8, 1.18-mm sieves for the three TMRs were also similar and averaged 9.7, 10.1, and 9.8; 22.2, 22.1, and 20.4; 34.4, 34.2, and 36.9%, respectively. The values of pef_m_, pef_>8_, and pef_>1.18_ of the treatments were different among the systems. However, regardless of the methods and evaluation system, as well as the geometric mean and its standard deviation, their values were similar within each system. The peNDF values (% of DM) were similar in all treatments (Table 2).

### Intakes and apparent total-tract nutrient digestibility

Dry matter intake (*P =* 0.004), as well as NDF (*P =* 0.001), peNDF_m_ (*P =* 0.025), and peNDF_>1.18_ (*P =* 0.024) intake were significantly different, although the intake of CP, NFC, EE, and ash were similar among treatments. Replacing barley with BP increased DMI, but DMI in treatments B and BC were similar (Table 3). The nature of NFC affected DMI, as well as the production and composition of milk. Positive intake responses to feeding on BP were observed. Cows in the treatment BB group consumed more NDF, peNDF_m_, and peNDF_>1.18_ compared with those in treatments B and BC. The intake of starch (*P =* 0.002), sugars, and NDSC (*P =* 0.001) varied among the different NFC treatments. Cows fed on treatments B and BC showed a greater intake of starch compared with treatment BB. Cows assigned to the B diet showed the smallest sugar and NDSC intakes. NDF intake did not vary among treatments. However, there were some unexpected differences in the total NFC intake, ash, and CP. Digestibility of DM (*P =* 0.004), NDF (*P =* 0.004), NFC (*P =* 0.032), and EE (*P =* 0.03) varied among treatments, although digestibility of ADF, CP, and OM remained the same (Table 3). The highest DM, NDF, and EE and significantly lower NFC digestibility were observed in treatment BB.

### Rumen fermentation and kinetics

Lower pH levels were observed in cows that received treatments B and BC compared with those in treatment BB (*P =* 0.003). In addition, the NH_3_-N concentration varied among treatments (*P =* 0.035). The NH_3_-N concentrations were significantly lower in treatments B and BC than in treatment BB. The total VFA concentration in treatment BC was similar to those observed in treatments B and BB. Acetate concentration levels were also significantly different (*P* < 0.001) among treatments. The lowest values of acetate and acetate: propionate ratio was observed in treatment B, whereas the highest were observed in treatment BB. The butyrate concentration remained the same among treatments. The lowest values for propionate, isobutytate, valerate, and isovalerate were observed in treatment BB, whereas the highest were in treatment B.

The ruminal particulate passage rate was highest and lowest in treatment BC and BB, respectively. The ruminal and total mean retention time were higher in treatments BB than in treatments B and BC. Contrary to the passage rate, treatments BB and BC showed the highest and lowest ruminal mean retention times, respectively. However, total mean retention time in treatment BB was higher than in treatments B and BC (Table 4). The time delay of the marker was significantly lower in treatment B than in BC (Table 4).

**Table 4:** Ruminal metabolites and kinetic of digestion of three total mixed rations containing three different sources of non-fiber carbohydrate

**Item**	**Treatments**^**1**^			**SEM**	***P *** **value**
	**B**	**BC**	**BB**		
pH	6.62^c^	6.73^b^	6.83^a^	0.002	0.003
NH_3_-N^2^, mg/dL	6.76^b^	6.68^b^	7.71^a^	0.111	0.035
VFA concentration					
Total, mmol/L	125.44^a^	121.21^b^	125.01^a^	0.650	<0.001
Acetate, mol/100 mol	58.21^c^	60.21^b^	63.05^a^	0.435	<0.001
Propionate, mol/100 mol	26.75^a^	25.45^b^	23.04^c^	0.121	<0.001
Butyrate, mol/100 mol	6.48	6.33	6.28	0.104	0.075
Isobutytate, mol/100 mol	2.39^a^	2.29^a^	2.11^b^	0.093	0.023
Valerate, mol/100 mol	3.47^a^	3.35^ab^	3.25^b^	0.096	<0.001
Isovalerate, mol/100 mol	2.49^a^	2.37^ab^	2.27^b^	0.073	0.014
Acetate: propionate	2.16^c^	2.37^b^	2.75^a^	0.053	<0.001
Ruminal passage rate, %/h	4.36^b^	5.46^a^	4.06^c^	0.097	0.012
Ruminal mean retention time, h	23.48^b^	18.32^c^	24.63^a^	0.871	0.002
Total mean retention time, h	54.36^b^	52.64^b^	61.35^a^	1.214	0.003
Time delay, h	9.25^b^	10.24^a^	9.86^ab^	0.444	0.021

^a, b, c^Means within a row with different subscripts differ (*P* < 0.05).

^1^Treatments were diets containing barley + basal diet (B), barley and corn + basal diet (BC), and barley and beet pulp + basal diet (BB).

^2^NH_3_-N ^=^ ammonia nitrogen concentration.

### Eating, ruminating, and chewing times

No differences in eating time were observed among treatments, although rumination time (*P* ≤ 0.001) and total chewing activity (*P* <0.001) varied (Table 5). Rumination time and total chewing activity were lower and higher in BC compared with that observed in B and BB. Treatment BB showed the highest rumination time and total chewing activity. Eating time (min/kg) per NDF intake, peNDF_m_, and peNDF_>8_ were higher in treatment B compared with treatments BC and BB. However, no significant differences with regard to eating time per NDF intake, peNDF_m_, and peNDF_>8_ were observed among treatments B and BB. Rumination time (min/kg) per DMI, NDF intake, peNDF_m_, peNDF_>8_, peNDF_>1.18_, and NFC were higher in treatments BB and BB compared with treatment BC. However, no significant differences on rumination time per DMI, NDF intake, peNDF_m_, peNDF_>8_, peNDF_>1.18_, and NFC were observed among treatments BB and BB. In addition, total chewing activity (min/kg) per DMI, NDF intake, peNDF_m_, peNDF_>8_, peNDF_>1.18_, and NFC were higher in treatment BB compared with treatments BC and BB. However, no differences on total chewing activity per peNDF_m_, peNDF_>8_, peNDF_>1.18_ were observed between treatments BC and BB, and the total chewing activity per DMI, NDF and NFC intake was lower in treatment BC compared with treatment BB. The eating time, rumination time, and total chewing activity per intake starch was higher in treatment BB than in treatments B and BC, whereas that of treatments B and BC were similar. Also, the eating time, rumination time, and total chewing activity per intake NDSC was lower in treatment BB than in treatments B and BC, and was significantly lower in treatment BC than in treatment B (Table 5).

**Table 5:** Chewing activity of cows fed on three total mixed rations of three total mixed rations containing three different sources of non-fiber carbohydrates

**Items**	**Treatments**^**1**^			**SEM**	***P *** **value**
	**B**	**BC**	**BB**		
Eating, min/d	257.8^a^	232.8^b^	249.4^ab^	10.272	0.045
Rumination, min/d	338.9^b^	286.1^c^	356.1^a^	7.430	<0.001
Total chewing activity, min/d	596.7^a^	518.9^b^	605.5^a^	10.482	<0.001
Chewing behaviour per different nutrients, min/kg					
Eating					
DMI	12.19	11.08	11.174	0.552	0.200
NDF	33.70^a^	30.00^b^	29.10^b^	0.972	0.004
peNDF_m_^2^	50.55^a^	45.29^b^	43.45^b^	2.430	0.033
peNDF _> 8_^3^	97.93^a^	86.75^b^	92.97^ab^	4.431	0.002
peNDF _> 1.18_^4^	50.85	45.20	43.37	4.294	0.063
NFC	31.36	28.46	29.42	1.154	0.088
Starch	54.97^b^	54.78^b^	76.98^a^	4.452	<0.001
NDSC^5^	89.52^a^	69.70^b^	55.55^c^	3.762	<0.001
Rumination					
DMI	16.03^a^	13.61^b^	15.95^a^	0.350	<0.001
NDF	44.30^a^	36.87^b^	41.55^a^	1.561	<0.001
peNDF_m_	66.45^a^	55.66^b^	62.04^a^	1.902	0.003
peNDF _> 8_	128.74^a^	106.61^b^	132.74^a^	5.242	0.009
peNDF _> 1.18_	66.84^a^	55.55^b^	61.93^a^	1.615	0.002
NFC	41.23^a^	34.98^b^	42.09^a^	0.812	<0.001
Starch	72.26^b^	67.32^b^	109.91^a^	6.692	<0.001
NDSC	117.67^a^	85.66^b^	79.31^c^	3.833	<0.001
Total chewing activity					
DMI	28.23^a^	24.69^c^	26.13^b^	0.671	<0.001
NDF	78.00^a^	66.87^c^	68.06^b^	2.031	<0.001
peNDF_m_	117.00^a^	100.95^b^	101.61^b^	3.292	<0.001
peNDF _> 8_	216.41^a^	193.35^b^	225.71^a^	15.320	<0.001
peNDF _> 1.18_	117.69^a^	100.76^b^	101.43^b^	3.462	0.006
NFC	72.59^a^	63.43^c^	68.94^b^	1.752	<0.001
Starch	127.23^b^	122.09^b^	186.88^a^	7.860	<0.001
NDSC	207.19^a^	155.36^b^	134.86^c^	6.744	<0.001

^a, b, c^Means within a row with different subscripts differ (*P* < 0.05).

^1^Treatments were diets containing barley + basal diet (B), barley and corn + basal diet (BC), and barley and beet pulp + basal diet (BB).

^2^peNDF_m_ = physically effective NDF, based on DM retained on sieve 1.18 mm [13].

^3^peNDF_>8_ = physically effective NDF, determined as the proportion of DM retained on sieves of the original version of Penn State particle separator [10].

^4^peNDF_>1.18_ = physically effective NDF, determined as the proportion of DM retained on sieves of the new version of Penn State particle separator [11].

^5^NDSC = Neutral detergent soluble carbohydrate.

### Milk production

Although milk yield was not altered, replacing barley with corn and BP in the diet resulted in an increase in milk fat [yield (*P* = 0.013)] and percentage (*P* <0.001) and a decrease in milk protein (yield (*P* = 0.021) and percentage [(*P* = 0.001); Table 6)], respectively.

**Table 6:** Milk production and composition of cows fed on three total mixed rations containing three different sources of non-fiber carbohydrates

**Item**	**Treatments**^**1**^			**SEM**	***P *** **value**
	**B**	**BC**	**BB**		
Yield of milk and milk components, kg/d					
Milk	25.11	25.50	25.83	0.653	0.062
3.5% FCM	21.69	22.43	22.69	0.645	0.070
Fat	0.86^b^	0.90^a^	0.91^a^	0.033	0.013
Protein	0.81^a^	0.80^b^	0.80^b^	0.025	0.021
Lactose	1.17	1.20	1.16	0.020	0.062
Total solid	3.47	3.42	3.57	0.060	0.076
Milk composition, %					
Fat	3.43^b^	3.55^b^	3.63^a^	0.087	<0.001
Protein	3.23^a^	3.15^b^	3.10^b^	0.030	0.001
Lactose	4.67	4.69	4.66	0.045	0.058
Total solid	11.93	11.89	12.01	0.098	0.060

^a, b^Means within a row with different subscripts differ (*P* < 0.05).

^1^Treatments were diets containing barley + basal diet (B), barley and corn + basal diet (BC), and barley and beet pulp + basal diet (BB).

## Discussion

### Nutrient composition, particle size distribution, and physical effectiveness

The pef_>8_ values were lowest for TMRs, although the pef_m_ and pef_>1.18_ values were higher and very close. In contrast to the peNDF_m_ and peNDF_>1.18_, the peNDF_>8_ values were similar among treatments. In this experiment, peNDF_m_ and peNDF_>1.18_ were higher than 21% of the DM that Mertens [13] suggested to adequately stimulate chewing activity that was required to maintain an average ruminal pH of greater than 6.0. The peNDF content of experimental rations was adequate and the differences among treatments were attributable to the effects of NFC sources or its nature. The concentration of starch was similar in the B and BC treatments and higher than that observed in the BB treatment, but the NDSC significantly varied among treatments. These results thus confirm that intake of starch and NDSC varied with different NFC treatments. We hypothesized that the use different digestion products, which are the NFC complement of the diet, would alter feed intake, ruminal digestion kinetic, chewing activity, and the production and composition of milk.

### Intake and apparent total-tract nutrient digestibility

The intake of peNDF_m_ and peNDF_>1.18_ did not follow the same trend as the pef content of the diets, although this was similar to that of NDF; however, this increased in treatment BB. Treatment BB, which contained more NSDC, resulted in a significant increase in DMI. These results have been confirmed by several previous studies. Substitution of sugars and NDSC with starch has increased [19-22] or did not change [23] the intake in dairy cattle. Increases in dietary concentrations of NDSC at the expense of starch also increased [20,24], decreased [22,25], or did not affect DMI [24]. Changes in the passage rate or fiber digestibility using various NFC sources may partial explain these changes [6]. A higher intake in treatment BB could have been expected based on the high NDSC and elevated starch content of barley and corn compared with treatments B and BC.

In this experiment, intake might have been regulated by the metabolism of propionate in the liver, and ruminal propionate production generally increases with a higher rate of starch fermentation. The amount of starch intake was 4.69, 4.25, and 3.24 kg/d, and ruminal molar percent of propionate in total VFA was 26.75, 25.45, and 23.04 mol/100 mol in treatments B, BC, and BB, respectively, showing that starch intake decreased with a higher rate of starch intake (Table 4). Therefore, it seems that propionate metabolism in the liver caused the reduction in DMI in treatments B and BC compared with BB because hepatic propionate oxidation probably decreased with the supplementation of BP [25]. In addition, ruminal pH might be implicated in the control of feed intake, particularly in satiety, because VFAs were more rapidly absorbed in the undissociated form at lower pH. In addition, substituting a high-fiber byproduct such as BP for grain may have resulted in the reduction of energy content of the ration or the limitation of intake by the physical filling effects of the diet.

The NFC sources such as BP have a similar range of NDF content as forages, although its particle length was generally smaller. In several experiments, these sources were substituted with forage or grain in diets and then the DMI was measured [3,5,6,22]. When DMI is regulated by ruminal distension, its substitution with forage in diets results in an increase in DMI, whereas its substitution with grain decreases DMI [2,4,13]. However, non-forage fiber sources showed inconsistent effects on DMI when substituted with either forage or grain in the diets of lactating cows. In the current experiment, the physical characteristics of the diets were similar; therefore, it seems that these do not differ in their relative filling effects. In treatment BB, the DMI was not regulated by ruminal distension [3,25]. The greater concentration of propionate in the ruminal liquid in treatment B compared with treatments BC and BB suggests that stimulated propionate receptors in the ruminal region may have controlled satiety and DMI, because propionate is more hypophagic than acetate, resulting in a lower DMI in treatment B versus treatments BC and BB [19,26].

Partially replacing barley with BP in the dairy cow diet resulted in an increase in total digestibility of DM, NDF, and EE, and a decrease in the digestibility of NFC. However, partially replacing barley with corn in the dairy cow diets showed minimal effects on total digestibility. Unfortunately, total digestibility of starch was not measured in this study. In the present study, decreased fiber digestibility in treatment B using barley was consistent with the reduction in rumination time, ruminal pH, and acetate to propionate ratio. The consumption of diets rich in NDSC imparted positive effects on ruminal digestion. In addition, the results of this experiment might be influenced by differences in the digestible NDF of the treatment diet and not simply the source of NFC, as implied in the objectives of this study.

Differences in digestible NDF rations or starch, NDSC, and sugar content were associated with several parameters, especially acetate and propionate concentrations and their ratio in the rumen, as well as milk fat and protein content. These were significantly different among treatments (*P* < 0.001) and treatment BB showed the highest intake of potentially digestible fiber or NDSC. Previous studies have confirmed that animal intake response to supplemented starch- or pectin-rich feeds remained the same, although changes in digestibilities have been observed. Valk et al. [22] reported that cows fed on corn consumed more DM and produced more milk than cows fed on BP. However, DMI and digestibility of NDF were higher in cows fed on BP compared with those fed on corn meal.

The extent and rate of degradation is extremely variable among grains, byproducts, and different sources of NFC [6,27]. Variation in ruminal degradation of DM and OM across feeds has been reported to range from 29% to 90%, and 29% to 67%, respectively [27]. Total tract digestibility of corn ranges from 91.2% to 98.9%, depending on the processing method and grain type, with ground corn averaging 93.5% [28]. Ruminal degradation of starch in ground corn and ground barley ranged from 51% to 93%, and 42% to 91%, respectively [28]. In addition, ruminal degradation of DM and NDF in BP was 78% [29] and 94.1% [30], respectively. The potentially digestible fraction leaves the rumen either by degradation or by passage to the lower tract. Furthermore, feed characteristics and ruminal fractional passage rates would most likely vary; these tend to be higher with fine, non-forage fiber sources due to differences in ruminal retention time, which may be influenced by ruminal consistency [6]. The inclusion of BP in treatment BB resulted in a decrease in particulate passage rates, an increase in ruminal and total mean retention time, and a positive effect on ruminal and total digestion.

### Rumen fermentation and kinetics

Variables of ruminal pH such as the outflow rate of digesta from the reticulorumen or ruminal indicate that retention time influences total fiber degradation. Rapidly fermenting carbohydrates such as sugars, NDSC, and some starches have the potential to rapidly decrease ruminal pH by virtue of the sheer mass of organic acids produced in this digestive region within a relatively short period of time. The ruminal pH corresponded to a higher rate of degradation and fermentation of barley grain that produced the highest concentration of total VFA and propionate, lower acetate, and acetate: propionate ratio in treatment B compared with the other treatments (Table 4).

Beet pulp, which is rich in pectin or NDSC, has been shown to yield relatively high amounts of acetic acid, thus resulting in an increase in the acetate to propionate ratio and less butyrate and lactate compared with other carbohydrate sources [31]. In addition, using twelve Friesian cows and sixteen heifers, Lees et al. [21] reported that in wk 9 and 16, the ruminal VFA proportions of cows fed on BP were higher for acetate and butyrate and lower for propionate compared with cows fed on corn. The molar proportion of acetate in the current study and in previous investigations was probably altered by the tendency of BP to increase fiber intake and digestion. In contrast, Van Vuuren et al. [32] did not detect any differences in ruminal fluid pH or VFA concentrations in either experiment involving cows fed on corn products or BP and soybean hulls.

Changing the dietary source of NFC or NDSC and starch content of diet rations of lactating dairy cows has been shown to alter the ruminal environment and fermentation products. The effects, although not always consistent, have been observed on the proportions of VFA, the concentration of rumen NH_3_-N, and the extent of fiber digestion with feeding starch, sugars, or NDSC-rich feeds [6]. The application of treatment BC resulted in a higher acidic pH, lower total VFA and proportion of propionate, and increased acetate concentration and acetate:propionate ratio in the ruminal fluid. Replacing BP with barley resulted in a higher acidic pH, increased acetate concentration in ruminal fluid, without any change in total VFA, and decreased proportion of propionate, valerate, isobutyrate, and isovalerate. Therefore, compared with barley, BP created more favorable conditions for microbial utilization of other carbohydrates in the rumen, at least in part due to the neutral pH. Decreasing daily mean ruminal pH, which was associated with an increase in ruminal outflow rate of digesta, resulted in a decrease in ruminal NDF digestibility and total tract digestibility [2,8,9,33]. Zebeli et al. [9] showed a decrease in total tract NDF digestibility by increasing the outflow rate of particulate digesta, which was associated with a decrease in ruminal mat consistency. It might be possible that a low digesta passage rate and high consistency in the ruminal mat due to inclusion of BP in the treatment BB was associated with a higher water holding capacity, greater entrapment of small particles, decreased outflow rate of solid digesta, and increased ruminal NDF digestibility in dairy cows.

The ruminal particulate passage rate was highest and lowest in treatments BC and BB, respectively. Because corn has a higher specific gravity than barley and BB, inclusion of corn in the diet resulted in an increase in ruminal passage rate and a decrease in rumen mean retention time (Table 4), and consequently, it decreased rumination time and total chewing activity. In contrast, the higher total chewing activity in treatment BB might have resulted from the lower pH that was attributable to a high degradability of barley, which may have decreased the rate and extent of NDF digestion. In treatment BB, the BP did not result in a reduction in ruminal pH despite its high rate of NDF digestion because of the production of a higher concentration of acetate and lower propionate. However, it has a low functional specific gravity and a large particle size [34], which could decrease its passage rate and increase its ruminal mean retention time and consequently, rumination and total chewing activity.

Two characteristics that affect the passage of digesta are density and particle size. In addition, there is a clear negative relationship between particle density and ruminal mean retention time. Particles with a specific gravity of lower than the critical size (1.18 mm) quickly pass out of the reticulorumen [8]. The specific gravity for BP was 1.152, whereas that of barley and corn grain were higher than 1.237 [34]. Therefore, inclusion of BP in treatment BB decreased particulate passage rates and increased ruminal and total mean retention time. Particles with a density range of 1.2 to 1.5 showed the highest rate of passage in cattle and sheep [3,8,9]. Grain and its byproducts (except for BP and soy hulls] showed a higher rate of passage from the rumen than forages because of the higher functional specific gravity and a particle size much smaller than that associated with stimulation of rumination. Therefore, most grains would have a faster rate of passage from the rumen than forages and other byproducts.

In treatment B, negative associative effects resulting from a lower pH due to a high degradability of barley would probably decrease the rate of digestion. Beet pulp did not reduce ruminal pH despite its high rate of NDF digestion [3]; however, it has a low functional specific gravity and a large particle size [34], which could decrease its passage rate and increase its ruminal mean retention time. Therefore, BP should have a very high ruminal digestibility [29], potentially supporting levels of ruminal microbial protein synthesis and milk production, similar to that observed in corn [31]. However, generalization of these results under different feeding conditions would be an oversimplification because different dietary treatments can interact with each other, thus affecting passage rate.

### Eating, ruminating, and chewing times

Although the effect of forage particle size on chewing behavior has been substantially investigated, information on the effects of NFC, starch, or NDSC content of ration, especially when barley grain partially replaced with corn and BP, is limited. Despite the absence of differences in eating time, the rumination time and total chewing activity significantly varied among treatments. Prolonged total chewing time due to the increase in dietary peNDF resulted from an increase in ruminating time rather than an increase in eating time.

Treatment BB showed the highest rumination time and total chewing activity. Total daily chewing time varied from 8.6, 9.9, and 10.1 h, with ruminating time at 5.6, 4.8, and 5.9 h in treatments B, BC, and BB, respectively. These values were within limits previously reported for dairy cows [9,13], although the mean chewing time per kg DM ingested was less than 30 min, contrary to the recommendations of Mertens [13], who proposed values equal to or exceeding 30 min/kg DM intake as suitable for limiting the risk of digestive disorders. Eating time per day and per kilogram of DMI increased with the supplementation of BP. As diet NDF intake increased with added BP in the current study, a decrease in time eating per kilogram of NDF was observed. However, no effect of BP substitution for corn on time eating per day or per kilogram of DMI was reported [2,13]. Tafaj et al. [2] and Zebeli et al. [9] have previously shown that the fiber content (NDF or forage NDF) and non-fiber carbohydrates in the diet affected daily chewing and rumination activities. Forage content remained the same across treatments; therefore, the nature of NFC or starch and NDSC content impacted the rate of feed intake, ruminal mean retention time, rumination time, and total chewing activity.

### Milk production

Although milk yield was not altered, replacing corn and BP with barley in diets resulted in an increase in milk fat (yield and percentage), and a decrease in protein concentration (yield and percentage; Table 6). Dietary BP can increase [27], decrease [22], or support milk yield, which have also been observed in cows fed on corn or barley [22,35]. In addition, intake may be improved [21], decreased [22,35], or remain the same [27,35] using BP. Various dietary carbohydrates can result in changes in ruminal fermentation and generate VFAs patterns that influence the hormonal status of the animal and potentially affect milk production and composition.

The increase in fat secretion in treatments BC and BB has been shown to elevate both acetate and acetate: propionate ratio in the rumen. However, treatments BC and BB did not alter ruminal concentrations of either the total VFA or butyrate. Mansfield et al. [31] previously showed a higher milk fat concentration (3.82 vs. 3.64), whereas milk protein (3.01% vs. 2.90%) and yield were lower in cows fed on the BP diet compared with the corn diet. The fat content of milk was enhanced in treatment BB, which can be directly attributed to the increase in fiber intake and digestion in cows and the molar proportion of acetate in the rumen. Consistent with the current experiment, milk fat increased in cows fed on the BP diet as a replacement for grain [22,31] due to the higher fiber intake and greater acetate concentrations in the rumen. It seems that the higher milk fat observed in treatment BB was due to the increase in intake of digestible NDF and NDSC, ruminal mean retention time, rumination time, total chewing activity, ruminal acetate concentration, and ruminal pH.

A decrease in milk CP content and yield was observed when corn and BP were substituted with barley. This finding was confirmed by Mansfield et al. [31], but was in contrast to the findings of Valk et al. [22]. In contrast to the current experiment, a lower intake of CP, lower NH_3_-N concentrations in the rumen, and lower CP degradability for cows fed on BP was observed, compared with that in cows fed on corn [20] or barley [33]. The consumption of carbohydrates such as NDSC that are more extensively fermented in the rumen may improve the utilization of the NPN through the stimulation of microbial protein synthesis. In contrast, when NH_3_-N concentrations in the rumen cannot meet microbial requirements, the fermentation of fiber is reduced and the animal responds with a lower intake and digestibility. Total VFA concentration in treatments BC and BB were observed to decrease. The lower total rumen VFA is an indicator of lower ruminal fermentation of fiber that limited milk protein yield. Huhtanen [27], Rooke et al. [36], and Chcster-Jones et al. [20] previously showed that BP supports VFA concentrations in the rumen similar to those for corn and barley grain, whereas VFA concentrations decreased in some situations [6]. In addition, NFC sources that were used in the current experiment varied in their DM rate and extent degradability, support of microbial yield, and supply of protein to the small intestine because microbial CP yield is influenced by carbohydrate and nitrogen sources, rate of carbohydrate fermentation, bacterial growth rate, ruminal dilution rate, and pH [22].

## Conclusion

The results of this study indicate that changing dietary NFC or starch and NDSC that possess a different level of degradability may alter intake, ruminal mat consistency, chewing activity, ruminal environment, and retention time or passage rate, and lactation performance. However, studies on the optimal concentrations of NFC from different sources, in combination with other dietary components, are warranted to monitor the lactation performance of dairy cows.

## Competing interests

The authors declare that they have no competing interests.

## Authors’ contributions

All authors have made substantial contributions to: the research design, analysis or interpretation of data, and in drafting the paper. Both authors read and approved the final manuscript.
